# Giant Cell Arteritis Presenting as Recurrent Left Middle Cerebral Artery Territory Infarction

**DOI:** 10.7759/cureus.118208

**Published:** 2026-10-20

**Authors:** Katriye Kelly

**Affiliations:** 1 Internal Medicine, Mid Cheshire Hospitals NHS Foundation Trust, Crewe, GBR

**Keywords:** acute ischaemic stroke, corticosteroid treatment, giant cell arteritis (gca), temporal arteritis (ta), vasculitis

## Abstract

Giant cell arteritis (GCA) is the most common systemic vasculitis in older adults and may rarely present with cerebrovascular ischaemic events. We report a case of a woman in her 70s who developed recurrent left middle cerebral artery territory infarctions despite dual antiplatelet therapy. Following the recurrent stroke, further assessment revealed a history of temporal pain, scalp tenderness, headache, transient visual disturbance, and jaw discomfort, with markedly elevated inflammatory markers. Temporal and axillary artery ultrasonography performed after initiation of corticosteroid therapy was normal. Despite negative imaging findings, the overall clinical presentation was considered consistent with GCA-associated stroke. Treatment with high-dose corticosteroids resulted in complete resolution of cranial symptoms and no further cerebrovascular events. This case highlights the importance of considering GCA in older patients presenting with recurrent ischaemic stroke, particularly when accompanied by cranial symptoms and unexplained systemic inflammation. It also demonstrates that anterior circulation infarction may occur in GCA and that negative vascular ultrasonography does not exclude the diagnosis when clinical suspicion remains high.

## Introduction

Giant cell arteritis (GCA) is the most common systemic vasculitis affecting adults aged over 50 years and predominantly involves large- and medium-sized arteries, particularly the extracranial branches of the carotid circulation. Cerebrovascular ischaemia in GCA is primarily thought to result from inflammation and remodelling of the arterial wall, leading to stenosis or occlusion of the vessel and subsequent impairment of cerebral blood flow [1]. Untreated disease may result in serious ischaemic complications, including permanent visual loss and cerebrovascular events. Although stroke is a recognised complication of GCA, it remains relatively uncommon, occurring in approximately 4% of patients, with the majority involving the vertebrobasilar circulation [2-5]. Anterior circulation infarction, including middle cerebral artery (MCA) territory stroke, as discussed in this case report, is less frequently seen. GCA-related stroke can be difficult to distinguish from more common aetiologies of cerebral infarction, especially in older patients with conventional vascular risk factors [2-4]. Recognition of GCA-related stroke is clinically important as prompt initiation of high-dose corticosteroid therapy can prevent further ischaemic complications. However, diagnosis may be challenging when cerebrovascular events precede the development of classic cranial manifestations seen in GCA or when vascular imaging is non-diagnostic. We report a case of recurrent left MCA territory infarction despite dual antiplatelet therapy, in which GCA was established as the most likely diagnosis based on clinical findings, despite negative vascular ultrasonography. This case highlights that GCA should be considered a potential underlying cause of recurrent anterior circulation infarction, particularly when accompanied by classic cranial manifestations of the disease and marked systemic inflammation.

## Case presentation

A female patient in her 70s with a history of resistant hypertension, stable 3-mm anterior communicating artery aneurysm, and a previous transient ischaemic attack presented with acute-onset expressive dysphasia and a left-sided headache. Neurological examination demonstrated mild expressive dysphasia without motor or sensory deficits (National Institutes of Health Stroke Scale (NIHSS) score of 1) [6]. CT angiography demonstrated a short-segment occlusion of the proximal inferior M2 branch, with no significant internal carotid artery stenosis, arterial dissection, or aortic arch atheromatous plaque burden. The vertebral and basilar arteries were patent. MRI brain subsequently confirmed an acute left parietal infarction (Figures 1, 2). These findings were considered to provide an appropriate radiological correlate for the patient's presenting symptoms. She was commenced on dual antiplatelet therapy with aspirin and clopidogrel and discharged following rehabilitation and clinical improvement.

**Figure 1:**
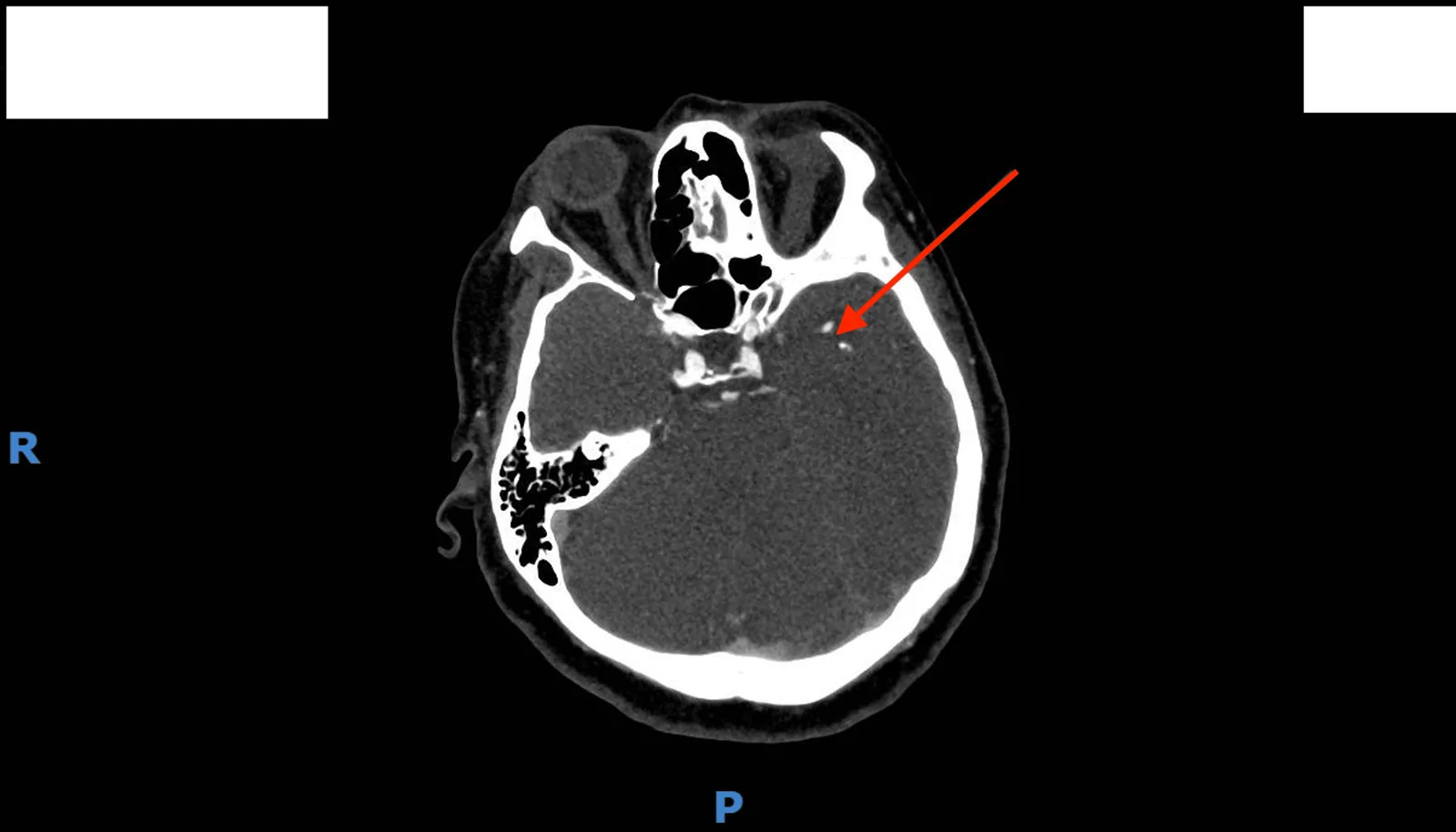
Axial contrast-enhanced CT angiographic image from an arch-to-vertex acquisition demonstrating a short-segment occlusion of the proximal inferior branch of the left M2 segment of the MCA (red arrow), with distal reconstitution. MCA: middle cerebral artery.

**Figure 2:**
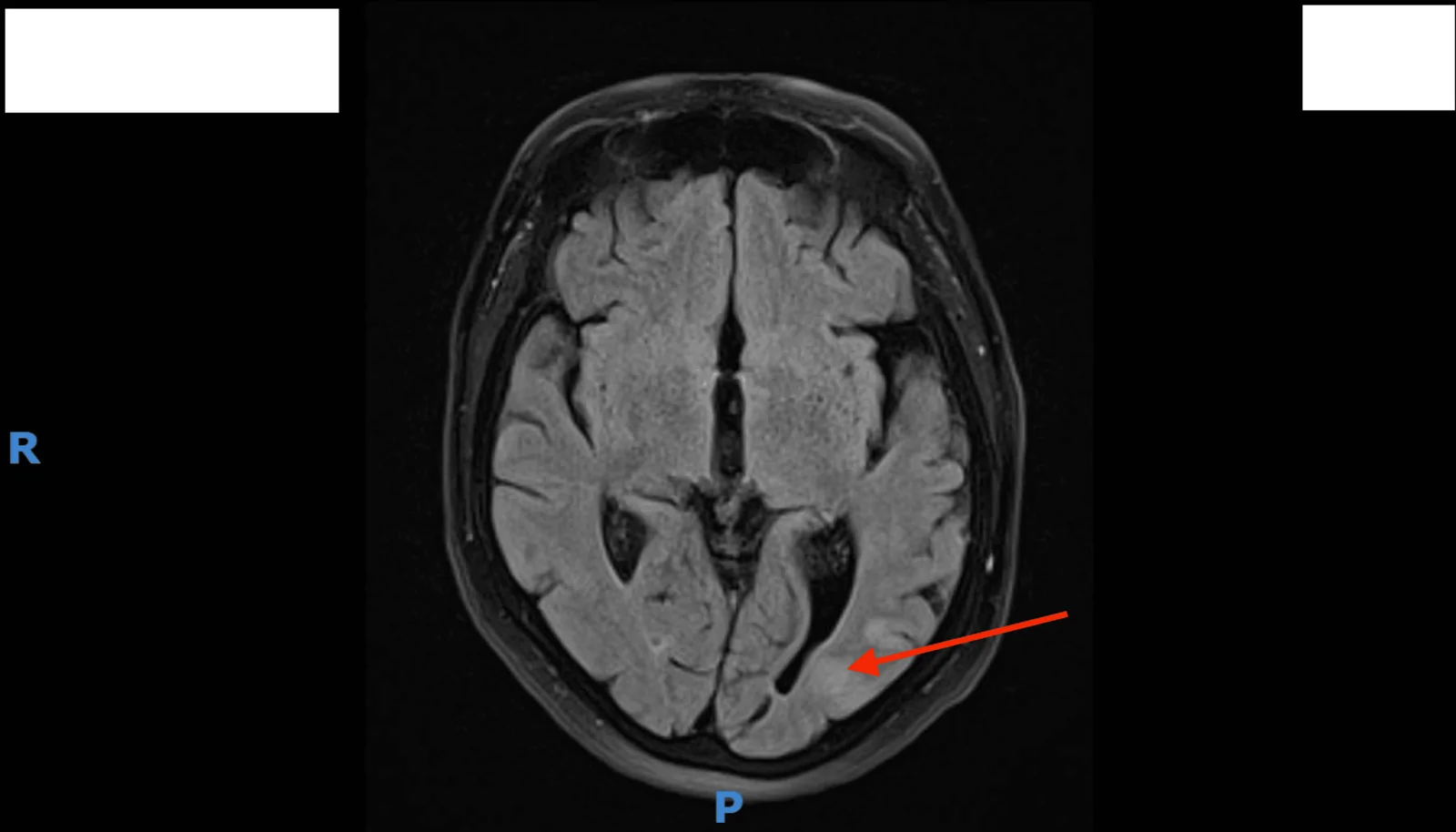
Axial T2-weighted fluid-suppressed MRI of the brain demonstrating hyperintense signal abnormality in the left parietal lobe (red arrow), corresponding to the left MCA territory infarction. MCA: middle cerebral artery.

Approximately one month later, she re-presented with a three-hour episode of recurrent aphasia, headache, bilateral blurred vision, and fatigue. MRI brain demonstrated two new acute infarcts within the left parietal lobe, anterior to the previous infarction, consistent with recurrent ischaemic stroke in the same vascular territory despite adherence to dual antiplatelet therapy (Figure 3). At this stage, recurrent infarction despite treatment raised concern for suspected treatment failure, and clopidogrel was replaced with ticagrelor. Cardioembolic investigations, including transthoracic echocardiography, carotid artery ultrasound, 72-hour cardiac monitoring, lipid screen, and HbA1c, were all unremarkable. 

**Figure 3:**
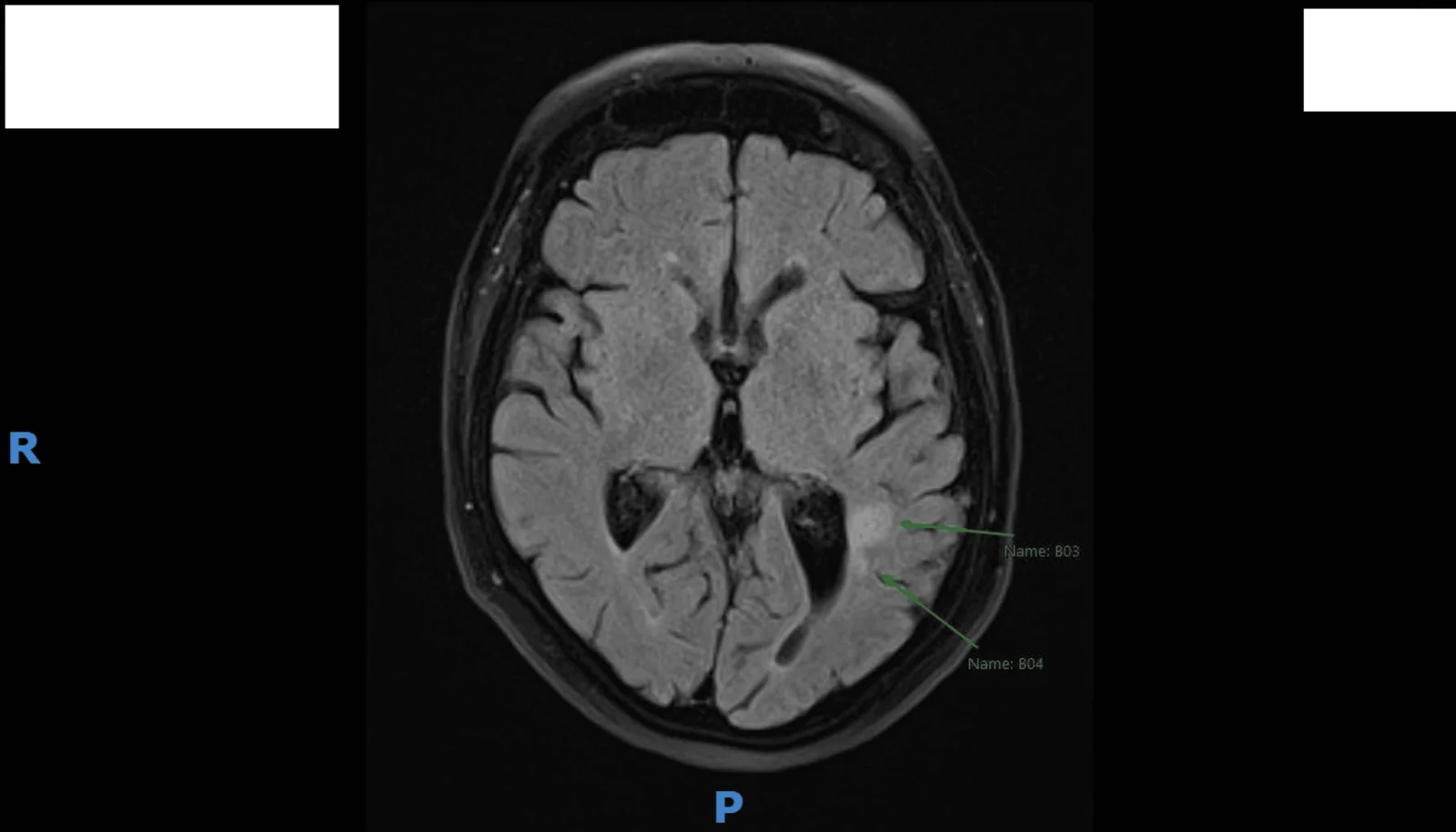
Axial T2-weighted fluid-suppressed MRI of the brain demonstrating two small hyperintense areas of infarction inferiorly within the left parietal lobe and left peritrigonal deep white matter (green arrows), located anterior to the previously demonstrated left occipital infarction.

Further history taking during her admission revealed several years of intermittent left-sided temporal and facial pain, with increasing frequency in the weeks preceding her recurrent stroke. She also reported scalp tenderness, headache, multiple previous episodes of transient visual disturbance, and jaw discomfort. This prompted us to start investigating GCA as a potential cause, with inflammatory markers subsequently coming back markedly elevated (ESR 101 mm/hour, CRP 135 mg/L) and deranged liver function tests (ALT 326 U/L).

Given the combination of recurrent ischaemic stroke, cranial symptoms, visual disturbance, and elevated inflammatory markers, a rheumatology review was sought and GCA was suspected. The ACR/EULAR classification criteria for GCA yielded a score of 9 (for age >50, jaw claudication, scalp tenderness, temporal headache, and ESR >50 mm/hour), supporting classification as GCA [7]. High-dose corticosteroid therapy was initiated with intravenous methylprednisolone at 500 mg once daily for three days, followed by oral prednisolone tapering starting at 60 mg for four weeks. An ophthalmological assessment was carried out, which showed normal visual acuity (logMAR −0.10 in the right eye and −0.02 in the left eye), pupils equal and reactive to light, normal extraocular motility, and no visual field defects. Optical coherence tomography (OCT) of the optic disc was unremarkable, with no evidence of arteritic anterior ischaemic optic neuropathy, central retinal artery occlusion, or retinal ischaemia. Temporal and axillary artery ultrasonography performed two months after corticosteroid initiation was normal (Figure 4). A PET-CT performed the following month showed no scintigraphic features to suggest active large-vessel vasculitis. A biopsy of the temporal artery was not considered appropriate as the patient had had an extensive period of corticosteroid treatment at this stage, with a good response.

**Figure 4:**
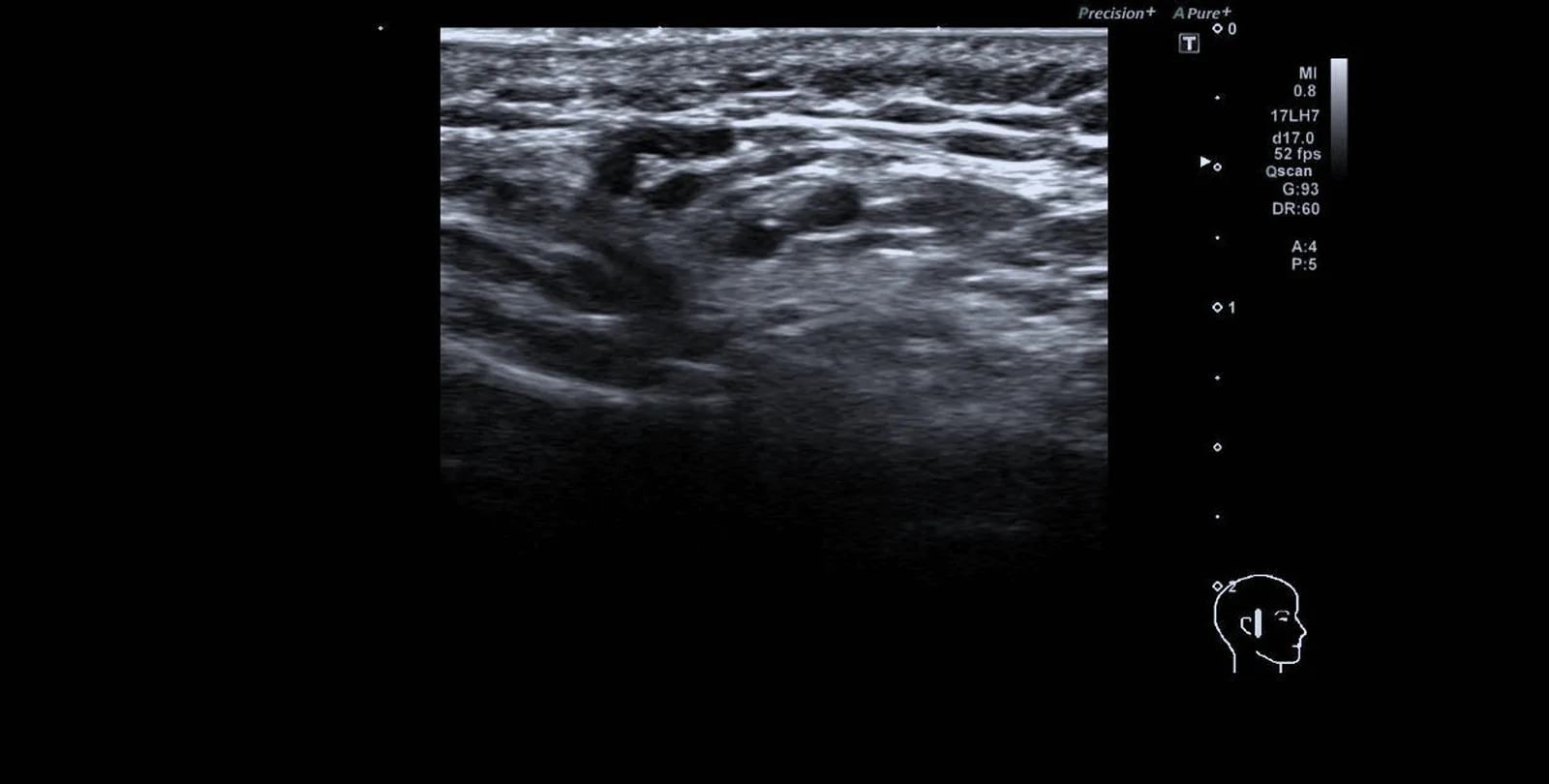
B-mode ultrasound of the superficial temporal artery demonstrating no sonographic evidence of active GCA. GCA: giant cell arteritis.

Despite the absence of definitive imaging or ophthalmological findings, the overall clinical picture was considered most consistent with GCA-associated recurrent ischaemic stroke. At rheumatology follow-up four weeks later, the patient reported complete resolution of the temporal pain and headaches, with no new visual disturbances. The inflammatory markers had started to normalise during this period, and the liver function tests had improved (Figure 5).

**Figure 5:**
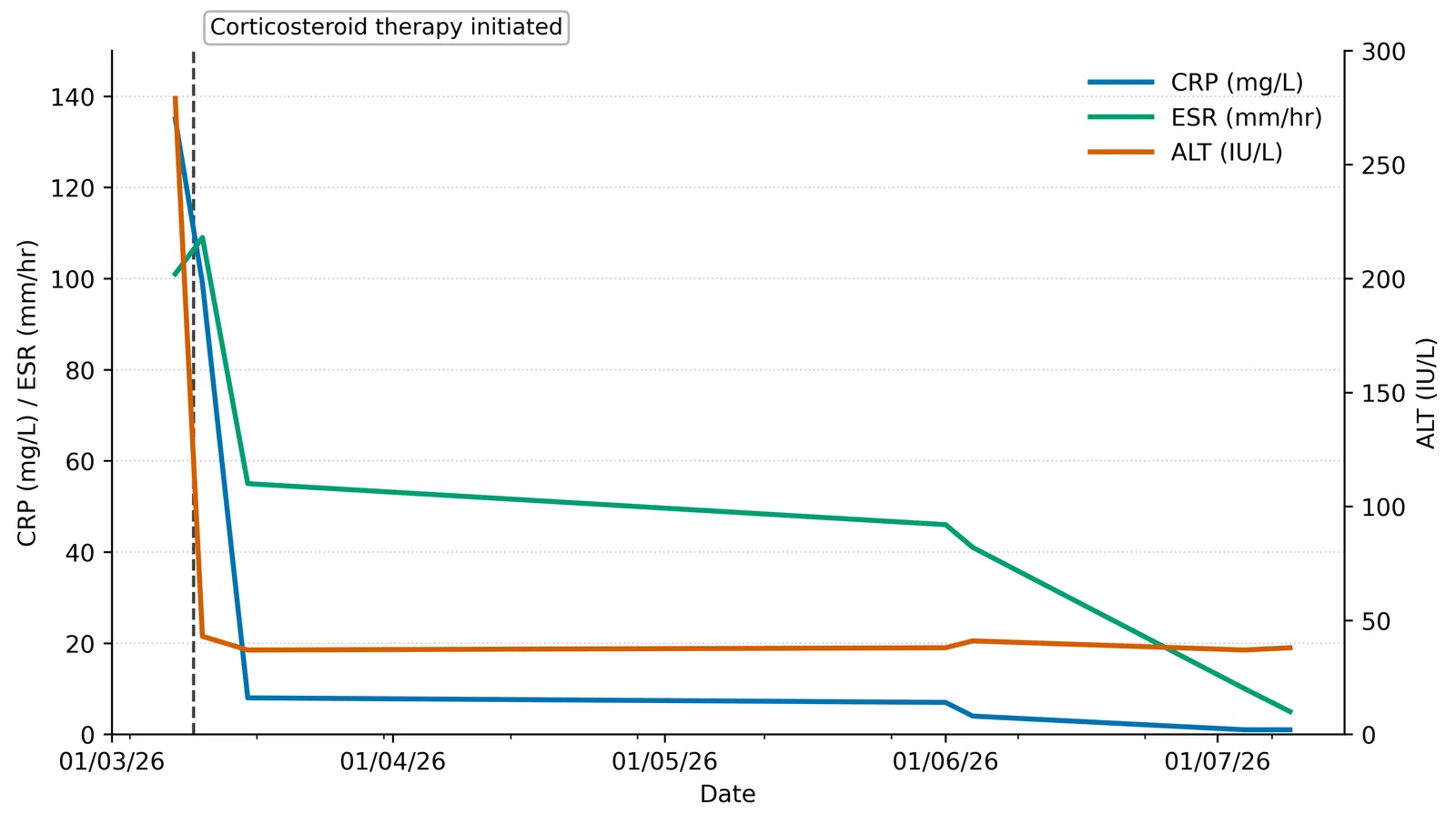
Serial inflammatory and hepatic biochemical markers during the clinical course. Trends in CRP, ESR, and ALT over the course of the patient's illness. The vertical dashed line indicates the initiation of corticosteroid therapy. CRP and ESR demonstrated a marked decline following the initiation of corticosteroid therapy, while ALT decreased substantially from its initial elevation and subsequently remained relatively stable. CRP: C-reactive protein; ESR: erythrocyte sedimentation rate; ALT: alanine aminotransferase.

Although there was no evidence of active disease on the PET scan or temporal and axillary artery ultrasonography, this was felt to be potentially influenced by prior corticosteroid treatment, and GCA remained the most probable diagnosis based on the wider clinical picture.

## Discussion

This case is notable for recurrent infarction within the left MCA territory despite adherence to dual antiplatelet therapy. This pattern differs from the more typical posterior circulation strokes reported in GCA. It has been demonstrated that vertebrobasilar involvement predominates among patients with GCA-related cerebrovascular events, with vertebral and large-vessel involvement more frequently seen in affected patients [2]. Similarly, a recent case series described six cases of stroke associated with temporal arteritis, highlighting that although stroke is an uncommon complication of GCA, it can still be the presenting manifestation of active disease [8]. Several additional case reports have described posterior circulation infarctions, including cerebellar and lateral medullary strokes, as initial presentations of GCA, further emphasising the recognised association between GCA and vertebrobasilar ischaemia [9,10]. The occurrence of recurrent MCA territory infarction in our patient therefore represents a much less common pattern of cerebrovascular involvement.

The diagnosis in this patient was challenging for several reasons, one being that the patient already had multiple vascular risk factors and a previous transient ischaemic attack, making recurrent atherosclerotic or embolic stroke a plausible alternative explanation. On re-presentation, recurrent infarction despite being on dual antiplatelet therapy raised concern regarding treatment failure. However, further history taking revealed several characteristic cranial symptoms (temporal pain, scalp tenderness, headache, transient visual disturbance, and jaw discomfort), which were also accompanied by markedly elevated inflammatory markers. This highlights the importance of thorough history taking in patients, as ultimately it was the presence of these features that led to the consideration of GCA being the cause rather than a purely thromboembolic mechanism.

An additional diagnostic challenge was the absence of supportive vascular ultrasound findings. Temporal artery ultrasonography is an important diagnostic tool in suspected GCA, particularly when characteristic inflammatory changes are demonstrated. However, it is important to note that a negative study does not exclude the diagnosis, especially when performed after corticosteroid therapy has already been commenced. Chandrashekhar et al. described the role of temporal artery ultrasonography in GCA diagnosis while demonstrating that imaging findings must be interpreted alongside the clinical presentation [11]. In our case, ultrasonography was performed after the patient had already received almost two months of high-dose corticosteroids, which may have reduced the sensitivity of the examination and contributed to the absence of sonographic abnormalities.

Several published cases demonstrate that GCA-related strokes may occur before the diagnosis of vasculitis or in the absence of classic cranial manifestations. Hiyama et al. reported recurrent cerebellar infarctions in a patient with atypical GCA symptoms, where the diagnosis was established only after consideration of inflammatory markers and the broader clinical picture [12]. Kargiotis et al. described acute ischaemic strokes associated with diffuse intracranial stenoses due to GCA, demonstrating that cerebrovascular involvement may result from inflammatory arterial narrowing beyond the extracranial vessels traditionally associated with the disease [13]. Both these reports highlight the importance of considering GCA in older patients with recurrent or unexplained cerebral infarction, particularly when imaging suggests an unusual vascular pattern or when inflammatory markers are disproportionately elevated in the clinical context.

The mechanism underlying GCA-associated stroke remains incompletely understood but is thought to involve inflammatory arterial wall injury, progressive luminal narrowing, thrombosis, and potentially vasospasm. Kawamura et al. described recurrent ischaemic stroke caused by vasospasm associated with GCA, suggesting that active vascular inflammation may contribute to recurrent cerebral ischaemia despite conventional antithrombotic therapy [14].

The favourable response to corticosteroid therapy was consistent with the working diagnosis of probable GCA. Following treatment, the patient's headache, scalp tenderness, temporal pain, and visual symptoms resolved completely, and no further cerebrovascular events have occurred thus far. However, the response to treatment alone is not diagnostic of GCA. The diagnosis was based on the overall clinical presentation, including the symptom profile, markedly elevated inflammatory markers that subsequently have started to normalise on the most recent blood test, and exclusion of alternative stroke mechanisms. Although recurrent visual disturbance formed part of the clinical presentation, ophthalmological assessment demonstrated no objective evidence of ocular ischaemia. The precise mechanism underlying these visual symptoms therefore remains uncertain and could not be directly attributed to either the cerebral infarctions or ocular involvement from GCA. The absence of positive ultrasonographic findings does not negate the diagnosis, as GCA remains a clinical diagnosis, supported by imaging and laboratory findings rather than excluded by a single negative investigation.

## Conclusions

Although GCA was not confirmed by imaging or biopsy, it remained the most likely diagnosis based on the overall clinical presentation. This case highlights the importance of considering GCA in older patients presenting with recurrent ischaemic stroke, particularly when accompanied by headache, scalp tenderness, jaw claudication, visual disturbance, or unexplained systemic inflammation. Although posterior circulation involvement remains the most frequently reported cerebrovascular manifestation, anterior circulation infarction may occur and can mimic more common stroke mechanisms. Early recognition through thorough history taking and prompt corticosteroid therapy remain essential to reduce the risk of further neurological and ophthalmological complications.
